# Alcohol SMART-ED: study design to examine the role of assessment reactivity in screening, brief intervention, and referral to treatment

**DOI:** 10.1186/1940-0640-7-S1-A77

**Published:** 2012-10-09

**Authors:** Dennis Donovan, Michael Bogenschutz, Harold Perl, Alyssa Forceheimes, Byron Adinoff, Raul Mandler, Neal Oden

**Affiliations:** 1 Alcohol & Drug Abuse Institute, University of Washington, Seattle, WA, USA; 2 Department of Psychiatry, University of New Mexico CASAA, Albuquerque, NM, USA; 3 Department of Prevention Research, National Institute on Drug Abuse, Bethesda, MD, USA; 4 Center on Alcoholism, Substance Abuse, and Addictions, University of New Mexico, Albuquerque, NM, USA; 5 Department of Psychiatry, University of Texas Southwestern Medical Center, Dallas, TX, USA; 6 Center for Clinical Trials Network, National Institute on Drug Abuse, Bethesda, MD, USA; 7 The EMMES Corporation, Rockville, MD, USA

## 

Questions have been raised in screening, brief intervention, and referral to treatment (SBIRT) research concerning the role the assessment process may serve in contributing to behavior change. However, most SBIRT trials have not been designed in a way to disaggregate the impact of assessment versus the combined effect of assessment plus brief intervention. We describe the design of the National Institute on Drug Abuse-Clinical Trials Network (NIDA-CTN) Screening, Motivational Assessment, Referral, and Treatment (SMART-ED) trial, with a focus on the screening and assessment instruments used and three treatment conditions to which participants have been randomized: minimal screen only (MSO); screening, assessment, and referral to treatment (if indicated) (SAR); or screening, assessment, and referral plus a brief intervention (BI) with two telephone follow-up booster calls (BI-B). The screening and assessment measures are relatively brief in the context of a clinical trial to minimize participant burden and assessment reactivity. The design allows a comparison of the impact of assessment as an independent factor over and above minimal screening (SAR versus MSO) as well as an evaluation of the incremental benefit of the BI with booster calls over and above assessment without BI (BI-B versus SAR). Assessment reactivity is of concern, especially in BI studies, because it may reduce effect size and conceal therapeutic benefit. On the other hand, if found to contribute independently to behavioral change, assessments could be designed to maximize the therapeutic benefit they provide. The design used in the SMART-ED trial will allow an evaluation of the independent and incremental contribution of the assessment process to behavior change.

